# Plant Growth-Promoting Bacteria: Mechanisms and Applications

**DOI:** 10.6064/2012/963401

**Published:** 2012-09-19

**Authors:** Bernard R. Glick

**Affiliations:** Department of Biology, University of Waterloo, 200 University Avenue South, Waterloo, ON, Canada N2L 3G1

## Abstract

The worldwide increases in both environmental damage and human population pressure have the unfortunate consequence that global food production may soon become insufficient to feed all of the world's people. It is therefore essential that agricultural productivity be significantly increased within the next few decades. To this end, agricultural practice is moving toward a more sustainable and environmentally friendly approach. This includes both the increasing use of transgenic plants and plant growth-promoting bacteria as a part of mainstream agricultural practice. Here, a number of the mechanisms utilized by plant growth-promoting bacteria are discussed and considered. It is envisioned that in the not too distant future, plant growth-promoting bacteria (PGPB) will begin to replace the use of chemicals in agriculture, horticulture, silviculture, and environmental cleanup strategies. While there may not be one simple strategy that can effectively promote the growth of all plants under all conditions, some of the strategies that are discussed already show great promise.

## 1. Introduction

There are currently around 7 billion people in the world and this is expected to increase to approximately 8 billion some time around the year 2020. When one considers both the expected worldwide population increase and the increasing environmental damage that is a consequence of ever greater levels of industrialization, it is clear that in the next ten to twenty years it will be a significant challenge to feed all of the world's people, a problem that will only increase with time. There is absolutely no time to lose; to feed this growing population, the world needs to begin to greatly increase agricultural productivity, and to do so in a sustainable and environmentally friendly manner. To feed the growing world, it is necessary to re-examine many of the existing approaches to agriculture that includes the use of chemical fertilizers, herbicides, fungicides, and insecticides. Instead, sustainable agriculture will likely make much greater use of both transgenic plants (for example, see http://www.isaaa.org/inbrief/default.asp) and plant growth-promoting bacteria, or PGPB [1].

It has been estimated that around “40% of deaths worldwide are caused by water, air, and soil pollution” and that “environmental degradation, coupled with the growth in world population, are (considered to be) major causes behind the rapid (global) increase in human disease” (http://www.sciencedaily.com/releases/2007/08/070813162438.htm). That is, as a consequence of both increasing population and industrialization, the earth's atmospheric, terrestrial, and aquatic systems are no longer sufficient to absorb and break down the increasing amount of waste that we produce. As a result, the environment is increasingly contaminated with a range of toxic metals and organic compounds [2–4]. Recognizing the nature and magnitude of the problem is an important first step. However, even if all environmental pollution were to cease tomorrow, it is still essential that all of the contaminated lands and waters be remediated. One way to address this problem is through the use of phytoremediation, the purposeful use of plants to take up and concentrate or degrade a wide range of environmental pollutants [5–8]. Moreover, the addition of PGPB to plants that are used in phytoremediation protocols typically makes the entire remediation process much more efficacious [3, 9, 10].

## 2. Plant Growth-Promoting Bacteria (PGPB)

Soil is replete with microscopic life forms including bacteria, fungi, actinomycetes, protozoa, and algae. Of these different microorganisms, bacteria are by far the most common (i.e., ~95%). It has been known for some time that the soil hosts a large number of bacteria (often around 10^8^ to 10^9^ cells per gram of soil) and that the number of culturable bacterial cells in soil is generally only about 1% of the total number of cells present [11]. However, in environmentally stressed soils the number of culturable bacteria may be as low as 10^4^ cells per gram of soil [12]. Both the number and the type of bacteria that are found in different soils are influenced by the soil conditions including temperature, moisture, and the presence of salt and other chemicals as well as by the number and types of plants found in those soils [13]. In addition, bacteria are generally not evenly distributed in soil. That is, the concentration of bacteria that is found around the roots of plants (i.e., in the rhizosphere) is typically much greater than in the rest of the soil. This is because of the presence of nutrients including sugars, amino acids, organic acids, and other small molecules from plant root exudates that may account for up to a third of the carbon that is fixed by a plant [14–17]. 

Regardless of the number of bacteria in a particular soil sample, the bacteria may affect plants in one of three ways. The interaction between soil bacteria and plants may be (from the perspective of the plant) beneficial, harmful, or neutral [18]. However, the effect that a particular bacterium has on a plant may change as the conditions change. For example, a bacterium that facilitates plant growth by providing either fixed nitrogen or phosphorus, compounds that are often present in only limited amounts in many soils, is unlikely to provide any benefit to plants when significant amounts of chemical fertilizer is added to the soil. In addition, it is possible for a particular bacterium to affect different plants disparately. Thus, for example, an IAA overproducing mutant of the bacterium *Pseudomonas fluorescens* BSP53a stimulated root development in blackcurrant cuttings while inhibiting the development of roots in cherry cuttings [19]. This observation may be interpreted as indicating that the blackcurrant cuttings contained a suboptimal level of IAA that was enhanced by the presence of the bacterium. On the other hand, with the cherry cuttings the IAA level was optimal prior to the addition of the bacterium and the additional IAA provided by the bacterium became inhibitory. Notwithstanding these caveats, it is usually a straightforward matter to decide whether a bacterium either promotes or inhibits plant growth. 

The bacteria that can promote plant growth, that is, PGPB, include those that are free-living, those that form specific symbiotic relationships with plants (e.g., *Rhizobia* spp. and *Frankia* spp.), bacterial endophytes that can colonize some or a portion of a plant's interior tissues, and cyanobacteria (formerly called blue-green algae). Notwithstanding the differences between these bacteria, they all utilize the same mechanisms. PGPB may promote plant growth directly usually by either facilitating resource acquisition or modulating plant hormone levels, or indirectly by decreasing the inhibitory effects of various pathogenic agents on plant growth and development, that is, by acting as biocontrol bacteria [20].

Historically, *Rhizobia* spp. were studied extensively, from physiological, biochemical, and molecular biological perspectives, before much interest was shown in trying to understand or utilize other PGPB to facilitate plant growth [21–23]. Thus, these early studies became a conceptual starting point for mechanistic studies of PGPB. However, since unlike *Rhizobia* spp., most PGPB fix no or only a limited amount of nitrogen, studies to better understand some of the mechanisms used by PGPB have addressed a wide range of different mechanisms [13, 20, 24].

### 2.1. Commercialization

Despite the, still, limited understanding of PGPB-plant interactions, a number of these bacteria are nevertheless used commercially as adjuncts to agricultural practice [1, 25]. Commercialized PGPB strains include *Agrobacterium radiobacter, Azospirillum brasilense, Azospirillum lipoferum, Azotobacter chroococcum, Bacillus fimus, Bacillus licheniformis, Bacillus megaterium, Bacillus mucilaginous, Bacillus pumilus, Bacillus* spp., *Bacillus subtilis, Bacillus subtilis* var. *amyloliquefaciens, Burkholderia cepacia, Delfitia acidovorans, Paenobacillus macerans, Pantoea agglomerans, Pseudomonas aureofaciens, Pseudomonas chlororaphis, Pseudomonas fluorescens, Pseudomonas solanacearum, Pseudomonas* spp., *Pseudomonas syringae, Serratia entomophilia, Streptomyces griseoviridis, Streptomyces* spp., *Streptomyces lydicus* and various *Rhizobia* spp. However, PGPB inoculated crops represent only a small fraction of current worldwide agricultural practice. 

For the more extensive commercialization of PGPB strains, a number of issues need to be addressed. These include (i) determination of those traits that are most important for efficacious functioning and subsequent selection of PGPB strains with appropriate biological activities; (ii) consistency among regulatory agencies in different countries regarding what strains can be released to the environment, and under what conditions genetically engineered strains are suitable for environmental use; (iii) a better understanding of the advantages and disadvantages of using rhizospheric versus endophytic bacteria; (iv) selection of PGPB strains that function optimally under specific environmental conditions (e.g., those that work well in warm and sandy soils versus organisms better adapted to cool and wet environments); (v) development of more effective means of applying PGPB to plants in various settings (e.g., in the field versus in the greenhouse); (vi) a better understanding of the potential interactions between PGPB and mycorrhizae and other soil fungi.

## 3. Direct Mechanisms

### 3.1. Facilitating Resource Acquisition

The best-studied mechanisms of bacterial plant growth promotion include providing plants with resources/nutrients that they lack such as fixed nitrogen, iron, and phosphorus. Many agricultural soils lack a sufficient amount of one or more of these compounds so that plant growth is suboptimal. To obviate this problem and obtain higher plant yields, farmers have become increasingly dependent on chemical sources of nitrogen and phosphorus. Besides being costly, the production of chemical fertilizers depletes nonrenewable resources, the oil and natural gas used to produce these fertilizers, and poses human and environmental hazards. It would obviously be advantageous if efficient biological means of providing nitrogen and phosphorus to plants could be used to substitute for at least a portion of the chemical nitrogen and phosphorus that is currently used.

#### 3.1.1. Nitrogen Fixation

In addition to *Rhizobia* spp., a number of free-living bacteria, for example *Azospirillum* spp., are also able to fix nitrogen and provide it to plants [26]. However, it is generally believed that free-living bacteria provide only a small amount of what the fixed nitrogen that the bacterially-associated host plant requires [27]. Nitrogenase (*nif*) genes required for nitrogen fixation include structural genes, genes involved in activation of the Fe protein, iron molybdenum cofactor biosynthesis, electron donation, and regulatory genes required for the synthesis and function of the enzyme. In diazotrophic (nitrogen fixing) bacteria, *nif* genes are typically found in a cluster of around 20–24 kb with seven operons encoding 20 different proteins. Because of the complexity of this system, genetic strategies to improve nitrogen fixation have been elusive. At one time, some scientists believed once *nif *genes were isolated and characterized, that it would be possible to genetically engineer improvements in nitrogen fixation. And, a few individuals argued that it might be possible to genetically engineer plants to fix their own nitrogen. Today, these ideas seem somewhat naïve. 

Since the process of nitrogen fixation requires a large amount of energy in the form of ATP, it would be advantageous if bacterial carbon resources were directed toward oxidative phosphorylation, which results in the synthesis of ATP, rather than glycogen synthesis, which results in the storage of energy in the form of glycogen. In one experiment, a strain of *Rhizobium tropici* was constructed with a deletion in the gene for glycogen synthase [28]. Treatment of bean plants with this engineered bacterium resulted in a significant increase in both the number of nodules that formed and an increase in the plant dry weight in comparison with treatment with the wild-type strain. This is one of the very few examples of scientists genetically modifying the nitrogen fixation apparatus of a bacterium and obtaining increased levels of fixed nitrogen. Unfortunately, while this mutant increased nodule number and plant biomass in the field, it does not survive well in the soil environment.

Oxygen is both inhibitory to the enzyme nitrogenase and is also a negative regulator of *nif *gene expression; however, it is required for *Rhizobium* spp. bacteroid respiration. To prevent oxygen from inhibiting nitrogen fixation while at the same time providing sufficient oxygen for the bacteroides within the nodule to respire, it is possible to introduce bacterial hemoglobin, which binds free oxygen. Following transformation of *Rhizobium etli* with a *Vitreoscilla* sp. (a gram negative bacterium) hemoglobin gene, at low levels of dissolved oxygen, the rhizobial cells had a two- to threefold higher respiratory rate than the nontransformed strain. In the greenhouse, following inoculation of bean plants with hemoglobin-containing *R. etli* the plants had 68% more nitrogenase activity than plants inoculated with wild-type *R. etli*. This difference led to a 25–30% increase in leaf nitrogen content and a 16% increase in the nitrogen content of the resultant seeds [29]. 

A small and localized rise in plant ethylene levels is often produced following the infection of legumes by *Rhizobium* spp. This increased ethylene concentration can inhibit subsequent rhizobial infection and nodulation [30]. Some rhizobial strains can increase the number of nodules that form on the roots of a host legume by limiting the rise in ethylene by synthesizing a small molecule called rhizobitoxine [31] that chemically inhibits the functioning of the enzyme ACC synthase, one of the ethylene biosynthetic enzymes. Alternatively, some rhizobial strains produce the enzyme ACC deaminase which removes some of the ACC (the immediate precursor to ethylene in plants) before it can be converted to ethylene [30]. The result of lowering the level of ethylene in legume hosts is that both the number of nodules and the biomass of the plant may be increased by 25–40% [32, 33]. In the field, approximately 1–10% of rhizobial strains naturally possess ACC deaminase [34] thus it is possible to increase the nodulation efficiency of *Rhizobia* strains that lack ACC deaminase by engineering these strains with *Rhizobia* ACC deaminase genes (and regulatory regions) isolated from other strains. In one instance, insertion of an ACC deaminase gene from *R. leguminosarum* bv. *viciae* into the chromosomal DNA of a strain of *Sinorhizobium meliloti* that lacked this enzyme dramatically increased both the nodule number and biomass of host alfalfa plants [33]. However, because of political/regulatory considerations, genetically engineered strains of *Rhizobia* are currently not acceptable for use in the field in most jurisdictions. This political/regulatory constraint notwithstanding, several commercial inoculant producers have already begun to screen/test their more recently isolated *Rhizobia* strains for active ACC deaminase.

#### 3.1.2. Phosphate Solubilization

Despite the fact that the amount of phosphorus in the soil is generally quite high (often between 400 and 1,200 mg kg^−1^ of soil) most of this phosphorus is insoluble and therefore not available to support plant growth. The insoluble phosphorus is present as either an inorganic mineral such as apatite or as one of several organic forms including inositol phosphate (soil phytate), phosphomonesters, and phosphotriesters [35]. In addition, much of the soluble inorganic phosphorus that is used as chemical fertilizer is immobilized soon after it is applied so that it then becomes unavailable to plants and is therefore wasted.

The limited bioavailability of phosphorus from the soil combined with the fact that this element is essential for plant growth means that the inability to obtain sufficient phosphorus often limits plant growth [36]. Thus, solubilization and mineralization of phosphorus by phosphate-solubilizing bacteria is an important trait in PGPB as well as in plant growth-promoting fungi such as mychorrizae [37, 38]. 

Typically, the solubilization of inorganic phosphorus occurs as a consequence of the action of low molecular weight organic acids such as gluconic and citric acid, both of which are synthesized by various soil bacteria [38–40]. On the other hand, the mineralization of organic phosphorus occurs through the synthesis of a variety of different phosphatases, catalyzing the hydrolysis of phosphoric esters [38]. Importantly, phosphate solubilization and mineralization can coexist in the same bacterial strain [41].

Unfortunately, because of variable results, the commercial application of phosphate-solubilizing PGPB has been quite limited. In fact, the most consistent positive effects of applying phosphate-solubilizing bacteria are seen when these bacteria are coinoculated with bacteria with other physiological capabilities such as N fixation, or with mycorrhizal or nonmycorrhizal fungi [42].

#### 3.1.3. Sequestering Iron

Despite the fact that iron is the fourth most abundant element on earth, in aerobic soils, iron is not readily assimilated by either bacteria or plants because ferric ion or Fe^+3^, which is the predominant form in nature, is only sparingly soluble so that the amount of iron available for assimilation by living organisms is extremely low [43]. Both microorganisms and plants require a high level of iron, and obtaining sufficient iron is even more problematic in the rhizosphere where plant, bacteria and fungi compete for iron [44, 45]. To survive with such a limited supply of iron, bacteria synthesize low-molecular mass siderophores (~400–1500 Da), molecules with an exceptionally high affinity for Fe^+3^ (*K*_*a*_ ranging from 10^23^ to 10^52^) as well as membrane receptors able to bind the Fe-siderophore complex, thereby facilitating iron uptake by microorganisms [46, 47]. At the present time, there are over 500 known siderophores; the chemical structures of 270 of these compounds have been determined [46].

The direct benefits of bacterial siderophores on the growth of plants have been demonstrated in several different types of experiments. For example, (i) several studies using radiolabeled ferric-siderophores as a sole source of iron showed that plants are able to take up the labeled iron [48–55]; (ii) mung bean plants, inoculated with the siderophore-producing *Pseudomonas* strain GRP3 and grown under iron-limiting conditions, showed reduced chlorotic symptoms and an enhanced chlorophyll level compared to uninoculated plants [56]; (iii) the Fe-pyoverdine complex synthesized by *Pseudomonas fluorescens* C7 was taken up by *Arabidopsis thaliana* plants, leading to an increase of iron inside plant tissues and to improved plant growth [57]. 

The provision of iron to plants by soil bacteria is even more important when the plants are exposed to an environmental stress such as heavy metal pollution. In this case, siderophores help to alleviate the stresses imposed on plants by high soil levels of heavy metals [58–62]. 

Plant iron nutrition can affect the structure of bacterial communities in the rhizosphere. For example, transgenic tobacco that overexpresses ferritin and accumulates more iron than nontransformed tobacco has less bioavailable iron in the rhizosphere [63]. As a consequence, the composition of the rhizosphere bacterial community differed significantly when compared to nontransformed tobacco lines.

### 3.2. Modulating Phytohormone Levels

Plant hormones play key roles in plant growth and development and in the response of plants to their environment [64]. Moreover, during its lifetime, a plant is often subjected to a number of nonlethal stresses that can limit its growth until either the stress is removed or the plant is able to adjust its metabolism to overcome the effects of the stress [65]. When plants encounter growth limiting environmental conditions, they often attempt to adjust the levels of their endogenous phytohormones in order to decrease the negative effects of the environmental stressors [66]. While this strategy is sometimes successful, rhizosphere microorganisms may also produce or modulate phytohormones under in vitro conditions [66] so that many PGPB can alter phytohormone levels and thereby affect the plant's hormonal balance and its response to stress [65].

#### 3.2.1. Cytokinins and Gibberellins

Several studies have shown that many soil bacteria in general, and PGPB in particular, can produce either cytokinins or gibberellins or both [67–72]. Thus, for example, cytokinins have been detected in the cell-free medium of some strains of *Azotobacter* spp., *Rhizobium* spp., *Pantoea agglomerans*, *Rhodospirillum rubrum*, *Pseudomonas fluorescens, Bacillus subtilis,* and *Paenibacillus polymyxa.* Moreover, plant growth promotion by some cytokinin- or gibberellin-producing PGPB has been reported [73–77]. However, a detailed understanding of the role of bacterially-synthesized hormones and how the bacterial production of these plant hormones is regulated is not currently available. Thus, much of what we believe to be the role of bacterially-produced cytokinins and gibberellins is based on our knowledge of plant physiological studies following the exogenous addition of purified hormones to growing plants. Finally, some strains of phytopathogens can also synthesize cytokinins. However, it appears that PGPB produce lower cytokinin levels compared to phytopathogens so that the effect of the PGPB on plant growth is stimulatory while the effect of the cytokinins from pathogens is inhibitory.

#### 3.2.2. Indoleacetic Acid

Although several naturally occurring auxins have been described in the literature, indole-3-acetic acid (indoleacetic acid, IAA) is by far the most common as well as the most studied auxin, and much of the scientific literature considers auxin and IAA to be interchangeable terms [78, 79]. IAA affects plant cell division, extension, and differentiation; stimulates seed and tuber germination; increases the rate of xylem and root development; controls processes of vegetative growth; initiates lateral and adventitious root formation; mediates responses to light, gravity and florescence; affects photosynthesis, pigment formation, biosynthesis of various metabolites, and resistance to stressful conditions [80, 81]. 

It has been known for more than 70 years that different IAA concentrations affect the physiology of plants in dramatically different ways. Plant responses to IAA vary from one type of plant to another, where some plants are more or less sensitive to IAA than other plants; according to the particular tissue involved, for example, in roots versus shoots (the optimal level of IAA for supporting plant growth is ~5 orders of magnitude lower for roots than for shoots); and as a function of the developmental stage of the plant. However, the endogenous pool of plant IAA may be altered by the acquisition of IAA that has been secreted by soil bacteria. In this regard, the level of IAA synthesized by the plant is important in determining whether bacterial IAA stimulates or suppresses plant growth. In plant roots, endogenous IAA may be suboptimal or optimal for growth [82] and additional IAA that is taken up from bacteria could alter the IAA level to either optimal or supraoptimal, resulting in plant growth promotion or inhibition, respectively. 

IAA synthesized by bacteria may be involved at different levels in plant-bacterial interactions. In particular, plant growth promotion and root nodulation are both affected by IAA. The role of IAA that was synthesized by the PGPB *Pseudomonas putida* GR12-2 in the development of canola roots was studied following the construction of an IAA-deficient mutant of this strain [83]. Seed inoculation with wild-type *P. putida* GR12-2 induced the formation of roots that were 35–50% longer than the roots from seeds treated with the IAA-deficient mutant and the roots from uninoculated seeds. On the other hand, inoculation of mung bean cuttings with a mutant of the same strain [84], which overproduces IAA, yielded a much greater number of shorter roots compared with controls [85]. This result was explained by the combined effect of auxin on growth promotion and inhibition of root elongation by ethylene [86]. The bacterial IAA that was incorporated by the plant stimulated the activity of the enzyme ACC synthase, resulting in increased synthesis of ACC [86], and a subsequent rise in ethylene that inhibited root elongation [87]. Overall, bacterial IAA increases root surface area and length, and thereby provides the plant has greater access to soil nutrients. In addition, bacterial IAA loosens plant cell walls and as a result facilitates an increasing amount of root exudation that provides additional nutrients to support the growth of rhizosphere bacteria.

Most *Rhizobium* strains that have been examined have been found to produce IAA [88] and several studies have suggested that increases in auxin levels in the host plant are necessary for nodule formation [89]. Thus, mutants of the bacterium *Bradyrhizobium elkanii* that had a decreased level of IAA synthesis induced fewer nodules on soybean roots than did the wild-type strain [90]. In addition, in nodules induced by low IAA-producing mutants of *Rhizobium* sp. NGR234, the IAA content was found to be lower than in nodules induced by the wild-type strain, supporting the idea that part of the IAA found in nodules is of prokaryotic origin and that this IAA facilitates nodulation [91]. 

#### 3.2.3. Ethylene

The plant hormone ethylene is one of the simplest molecules with biological activity. According to the Hebrew Bible, the prophet Amos was a “herdsman and a nipper of figs.” This statement is interpreted as indicating that as early as the ninth century B C E, an awareness existed that nipping or piercing figs produced ethylene gas thereby hastening the ripening process and making the figs sweeter. 

The plant hormone ethylene has a wide range of biological activities and is active at concentrations as low as 0.05 *μ*L/L although ripening fruit may have ethylene levels of ~200 *μ*L/L [92]. Ethylene can affect plant growth and development in a large number of different ways including promoting root initiation, inhibiting root elongation, promoting fruit ripening, promoting flower wilting, stimulating seed germination, promoting leaf abscission, activating the synthesis of other plant hormones, inhibiting *Rhizobia* spp. nodule formation, inhibiting mycorrhizae-plant interaction, and responding to both biotic and abiotic stresses [92]. The ethylene that is synthesized as a response to various stresses is called “stress ethylene” [92] and it describes the increase in ethylene synthesis that is typically associated with various environmental stresses including extremes of temperature; high light; flooding; drought; the presence of toxic metals and organic pollutants; radiation; wounding; insect predation; high salt; various pathogens including viruses, bacteria, and fungi [93]. The increased amount of ethylene that is formed in response to various environmental stresses can exacerbate some of the symptoms of the stress or it can lead to responses that enhance plant survival under adverse conditions. This seemingly contradictory behavior may be explained by a model wherein plants that are exposed to stress quickly respond by producing a small peak of ethylene that initiates a protective response by the plant, for example, transcription of genes encoding defensive proteins [65, 94]. If the stress persists or is intense, a second much larger peak of ethylene occurs, often several days later. This second ethylene peak induces processes such as senescence, chlorosis, and abscission that may lead to a significant inhibition of plant growth and survival. 

Following the discovery in soil bacteria of the enzyme 1-aminocyclopropane-1-carboxylate (ACC) deaminase [95], several studies indicated that this enzyme was a common feature of many PGPB [96, 97]. In addition, a model was formulated wherein the role of this enzyme in the facilitation of plant growth by PGPB was elaborated [98]. In this model, PGPB colonize the seed or root of a growing plant and, in response to tryptophan and other small molecules in seed or root exudates, the bacteria synthesize and secrete IAA [78, 83]. This bacterial IAA, together with endogenous plant IAA, can either stimulate plant growth or induce the synthesis of the plant enzyme ACC synthase that converts the compound *S*-adenosyl methionine to ACC, the immediate precursor of ethylene in all higher plants. A portion of the newly synthesized ACC is excluded from seeds or plant roots [99], taken up by the PGPB, and converted by the enzyme ACC deaminase to ammonia and *α*-ketobutyrate, compounds that are readily assimilated. As a direct consequence of this enzyme's activity, the amount of ethylene produced by the plant is reduced. Therefore, root or seed colonization by PGPB that synthesize ACC deaminase prevents plant ethylene levels from becoming growth inhibitory [20, 98]. In the short term, the main visible effect of seed or root inoculation with ACC deaminase-producing bacteria is the enhancement of plant root elongation; promotion of shoot growth is generally seen in longer term experiments [13, 100–107]. In addition, other processes such as nodulation of legumes and mycorrhizal establishment in the host plant induce local increases in ethylene content. As a result, by lowering the local ethylene content in these plants, ACC deaminase-producing bacteria can increase the extent of both rhizobial nodulation and mycorrhizal colonization, in various legumes such as pea, alfalfa, mung bean, and chickpea [32, 33, 107, 108] and cucumber [109], respectively.

## 4. Indirect Mechanisms

The ability of biocontrol bacteria to indirectly promote plant growth has been the source of considerable interest, both in terms of (i) developing an understanding of some of the underlying mechanisms used by the biocontrol bacteria and (ii) utilizing these bacteria commercially instead of chemical pesticides. In fact, these two objectives are largely complementary. That is, understanding the mechanisms that are employed by biocontrol bacteria should facilitate the subsequent efficacious use of these bacterial strains in an applied setting. 

### 4.1. Antibiotics and Lytic Enzymes

The synthesis of a range of different antibiotics is the PGPB trait that is most often associated with the ability of the bacterium to prevent the proliferation of plant pathogens (generally fungi) [110–115]. Many of these antibiotics together with their specificity and mode of action have been studied in detail, and some of these biocontrol strains have been commercialized. One problem with depending too much on antibiotic-producing bacteria as biocontrol agents is that with the increased use of these strains, some phytopathogens may develop resistance to specific antibitoics. To prevent this from happening, some researchers have utilized biocontrol strains that synthesize hydrogen cyanide as well as one or more antibiotics. This approach is effective because, while hydrogen cyanide may not have much biocontrol activity by itself, it appears to act synergistically with bacterially encoded antibiotics.

Some biocontrol bacteria produce enzymes including chitinases, cellulases, *β*-1,3 glucanases, proteases, and lipases that can lyse a portion of the cell walls of many pathogenic fungi. PGPB that synthesize one or more of these enzymes have been found to have biocontrol activity against a range of pathogenic fungi including *Botrytis cinerea*, *Sclerotium rolfsii*, *Fusarium oxysporum, Phytophthora* spp., *Rhizoctonia solani,* and *Pythium ultimum* [116–119].

### 4.2. Siderophores

Some bacterial strains that do not employ any other means of biocontrol can act as biocontrol agents using the siderophores that they produce. In this case, siderophores from PGPB can prevent some phytopathogens from acquiring a sufficient amount of iron thereby limiting their ability to proliferate [120, 121]. It has been suggested that this mechanism is effective because biocontrol PGPB produce siderophores that have a much greater affinity for iron than do fungal pathogens [122] so that the fungal pathogens are unable to proliferate in the rhizosphere of the roots of the host plant because of a lack of iron [123]. In this model, the biocontrol PGPB effectively out-compete fungal pathogens for available iron.

On the other hand, the growth of plants is generally not affected by the depletion of iron in the rhizosphere caused by the siderophores produced by biocontrol PGPB because most plants can grow at much lower iron concentrations than most microorganisms [123]. In addition, many plants can bind, take up and then utilize the biocontrol PGPB iron-siderophore complex [124, 125]. 

Experimental evidence that is consistent with the involvement of biocontrol PGPB siderophores in the suppression of fungal pathogen-caused plant disease comes from several different studies. For example, some studies have included the use of mutants that were defective in siderophore production and found that these strains were less effective than the wild-type strains at protecting plants against fungal pathogens [126–128]. On the other hand, one study observed that siderophore overproducing mutants were more effective at protecting plants against fungal pathogens [129]. 

### 4.3. Competition

Although it is difficult to demonstrate directly, some indirect evidence indicates that competition between pathogens and nonpathogens (PGPB) can limit disease incidence and severity. Thus, for example, abundant nonpathogenic soil microbes rapidly colonize plant surfaces and use most of the available nutrients, making it difficult for pathogens to grow. For example, in one series of experiments, researchers demonstrated that treatment of plants with the leaf bacterium *Sphingomonas* sp. prevented the bacterial pathogen *Pseudomonas syringae* pv. tomato from causing pathogenic symptoms [130].

### 4.4. Ethylene

Plants typically respond to the presence of phytopathogens by synthesizing stress ethylene that exacerbates the effects of the stress on the plant [92]. Thus, one way to decrease the damage to plants caused by a wide range of phytopathogens is to lower the plant's ethylene response [131]. The simplest way to do this is to treat plants (generally the roots or seeds are treated) with ACC deaminase-containing PGPB [98]. To date, this strategy has been shown, in greenhouse and growth chamber experiments, to lower the damage to cucumber, potato, castor bean, tomato, carrot, and soybean plants [132–136]. Importantly, these studies have tested several different phytopathogens including *Pythium ultimum*, *Fusarium oxysporum*, *Erwinia carotovora*, *Agrobacterium tumefaciens*, *Agrobacterium vitis*, *Sclerotium rolfsii*, and *Rhizoctonia solani*. In addition, transgenic plants that express a bacterial ACC deaminase are protected to a significant level against damage from various phytopathogens [137, 138]. Notwithstanding these potentially exciting results, the ability of ACC deaminase-containing PGPB to decrease the damage to plants from pathogens, in the field, has not been tested. This likely reflects a reluctance of many individuals to deal with the potentially difficult regulatory approval process for doing this sort of field testing.

### 4.5. Induced Systemic Resistance

PGPB can trigger a phenomenon in plants known as induced systemic resistance (ISR) that is phenotypically similar to the systemic acquired resistance (SAR) that occurs when plants activate their defense mechanisms in response to infection by a pathogenic agent [139]. ISR-positive plants are said to be “primed” so that they react faster and more strongly to pathogen attack by inducing defense mechanisms. ISR does not target specific pathogens. Rather, it may be effective at controlling diseases caused by different pathogens. ISR involves jasmonate and ethylene signaling within the plant and these hormones stimulate the host plant's defense responses to a range of pathogens [140]. ISR does not require any direct interaction between the resistance-inducing PGPB and the pathogen [141]. Besides ethylene and jasmonate, other bacterial molecules such as the *O*-antigenic side chain of the bacterial outer membrane protein lipopolysaccharide, flagellar proteins, pyoverdine, chitin, *β*-glucans, cyclic lipopeptide surfactants, and salicylic acid have all been reported to act as signals for the induction of systemic resistance.

## 5. Modulating the Effects of Environmental Stress

Under ideal circumstances, a large portion of a plant's growth and development may be thought of as proceeding in a more or less linear fashion over time [65]. However, in the field, the growth of plants may be inhibited by a large number of different biotic and abiotic stresses. These stresses include extremes of temperature, high light, flooding, drought, the presence of toxic metals and environmental organic contaminants, radiation, wounding, insect predation, nematodes, high salt, and various pathogens including viruses, bacteria and fungi. Therefore, as a consequence of these many different environmental stresses, plant growth is invariably lower than it would be in their absence. Moreover, during its life, a plant may be subjected to a number of nonlethal stresses that limit its growth until either the stress is removed or the plant is able to adjust its metabolism to overcome the stress. Thus, in practice, plant growth typically consists of periods of maximal growth interspersed with periods of various levels of growth inhibition. When they are added to plants, PGPB may employ any one or more of several different mechanistic strategies in an effort to overcome this growth inhibition. 

### 5.1. Ethylene

Most of the aforementioned environmental stresses result in the production of inhibitory levels of stress ethylene. As mentioned above when discussing the stress ethylene produced as a consequence of phytopathogen infection, high levels of ethylene and the damage that it causes may be at least partially avoided by employing ACC deaminase-containing PGPB [142]. Some of the abiotic stresses whose effects can be ameliorated in this way include temperature extremes [143], flooding [144], drought [145, 146], metals and metaloids [60, 61, 147–152], hypoxia [153], salt [154–165], and organic contaminants [150, 151, 166–168].

The above mentioned reports from all over the world indicating that numerous different ACC deaminase-containing PGPB can provide significant protection to plants from a range of abiotic stresses suggests that this technology is ready to be utilized commercially in the field and that this approach could make a significant impact on agricultural practice. However, given the reluctance in many jurisdictions to utilize bacteria in agriculture on a large scale, it is likely that ACC deaminase-containing bacteria are more likely to find their first large scale commercial uses as components of phytoremediation protocols, that is, the simultaneous use of bacteria and plants to remove metals and organic contaminants from the environment [3, 9].

### 5.2. IAA

There are several reports indicating that some PGPB that do not contain ACC deaminase are nevertheless able to protect plants against the deleterious effects of abiotic stresses. In the more recent scientific literature, it has been suggested that PGPB may help plants to overcome abiotic stresses by providing the plant with IAA that directly stimulates plant growth, even in the presence of otherwise inhibitory compounds [169–176].

In addition to the above mentioned reports, a large number of studies have suggested that the bacteria that most effectively protect plants against a wide range of different stresses produce both IAA and ACC deaminase [65, 177–179]. One model that describes how IAA and ACC deaminase synergistically promote plant growth may described as follows [20, 65, 178]: the amino acid tryptophan is excluded by plant roots and then taken up by PGPB bound to the roots, where it is converted into IAA. The bacterially produced IAA is secreted, taken up by plant cells and, together with the plant's pool of IAA stimulates an auxin signal transduction pathway, including various auxin response factors. As a consequence, plant cells grow and proliferate; at the same time, some of the IAA promotes transcription of the gene encoding the enzyme ACC synthase, thereby yielding an increased concentration of ACC and eventually ethylene (as catalyzed by the enzyme ACC oxidase since ACC is the immediate precursor of ethylene). Various biotic and abiotic stresses may also either increase the synthesis of IAA or stimulate the transcription of the gene for ACC synthase. In the presence of a bacterium that contains the enzyme ACC deaminase, some ACC may be taken up the PGPB bound to the plant, and degraded to ammonia and *α*-ketobutyrate. Thus, an ACC deaminase-containing PGPB acts as a sink for ACC with the consequence that, following an environmental stress, a lower level of ethylene is produced by the plant and the stress response of the plant is decreased. As the level of ethylene in a plant increases, the transcription of auxin response factors is inhibited [65, 180–182]. In the absence of bacterial ACC deaminase, by limiting transcription of auxin response factors, ethylene limits both cell growth and proliferation, and (important for plant survival) IAA stimulation of the synthesis of additional ethylene. In the presence of ACC deaminase, less ethylene is formed. Thus, when ACC deaminase is present, transcription of auxin response factors is not inhibited, and IAA can stimulate cell growth and proliferation without simultaneously causing a buildup of ethylene. Consequently, ACC deaminase both decreases ethylene inhibition of plant growth, and allows IAA to maximally promote plant growth, both in the presence and absence of plant stress.

### 5.3. Cytokinin

Cytokinins are compounds with a structure resembling adenine (Sakakibara 2006) that are named based on their ability to promote cytokinesis or cell division in plants. They are produced by plants, some yeast strains and by a number of soil bacteria, including PGPB [66, 68]. Transgenic plants that overproduce cytokinins, especially during periods of abiotic stress, are significantly protected from the deleterious effects of those stresses [183]. Unfortunately, there are not yet any definitive studies indicating whether bacterially-produced cytokinins can also protect plants from abiotic stresses. This would involve a detailed comparison of the biological activity of cytokinin-producing PGPB with cytokinin minus mutants of those bacteria.

### 5.4. Trehalose

Trehalose is a nonreducing disaccharide, an *α*, *α*-1,1-glucoside, consisting of two molecules of *α*-glucose, that is widely distributed in nature. It is found in bacteria, yeast, fungi, plants, insects, and invertebrates. High levels of trehalose can act as a protectant against several different abiotic stresses including drought, high salt, and extremes of temperature. Trehalose, a highly stable molecule that is resistant to both acid and high temperature and can form a gel phase as cells dehydrate, replacing water and, as a result, decreasing damage from drought and salt. In addition, trehalose can prevent some of the protein degradation and aggregation that often occurs under both high and low temperature stresses.

One way to confer drought (and other stress) tolerance onto plants is to treat the plants with PGPB that have been engineered to overproduce trehalose [184, 185]. Thus, when bean plants were treated with the symbiotic bacterium *Rhizobium etli* that had been genetically engineered to overproduce trehalose, the host plants had more nodules, fixed more nitrogen, had more biomass and recovered to a greater extent from drought stress than plants inoculated with wild-type *R. etli* [185]. Similarly, when maize plants were treated with the PGPB *Azospirillum brasilense* that had previously been modified to overproduce trehalose, the treated plants were more drought resistant and produced more biomass than plants treated with wild-type *A. brasilense* [184]. Although it is also possible to engineer plants to overproduce trehalose, it is much simpler to use genetically manipulated PGPB to achieve the same end. In addition, a single engineered bacterial strain may effectively protect a large number of different crop plants.

### 5.5. Antifreeze

To function effectively in the field, a PGPB must be to persist and proliferate in the environment [186]. In addition, in cold and temperate climates many fungal phytopathogens are most destructive when the soil temperature is low. In those environments, cold tolerant (psychrotrophic) biocontrol PGPB are likely to be more effective in the field than mesophilic biocontrol strains. Moreover, in countries such as Canada, Sweden, Finland, and Russia, PGPB must be functional at the cool soil temperatures that are common in the spring (i.e., ~5–10°C). It would also be advantageous if added PGPB were able to survive repeated freeze thaw cycles that are common during the winter in many places. Nearly twenty years ago, several workers reported for the first time that some psychrophilic and psychrotrophic bacteria, including PGPB, secrete antifreeze proteins into the surrounding medium when the bacteria are grown at low temperatures [187–189]. Bacterial antifreeze proteins, some of which may also have ice-nucleation activity, appear to regulate the formation of ice crystals outside of the bacterium, thereby protecting the bacterial cell wall and membrane from potentially lethal damage (piercing) from the formation of large ice crystals that might otherwise occur at freezing temperatures. 

Since the initial reports of bacterial antifreeze proteins, there have been several additional reports documenting the isolation and characterization of bacterial antifreeze proteins [155, 190–195]. However, none of these studies have explored the possibility of using this activity to facilitate the functioning of PGPB in environments that include cold temperatures.

## 6. Conclusions

The use of PGPB as an integral component of agricultural practice is a technology whose time has come. These bacteria are already being used successfully in a number of countries in the developing world and this practice is expected to grow. In the more developed world, where agricultural chemicals remain relatively inexpensive, the use of PGPB occupies a small but growing niche in the development of organic agriculture. In addition, it is reasonable to expect the increased use of PGPB in various phytoremediation strategies.

However, the more widespread utilization of PGPB will necessitate that a number of issues be addressed. In the first instance, going from laboratory and greenhouse experiments to field trials to large scale commercial field use will require a number of new approaches for the growth, storage, shipping, formulation and application of these bacteria. Second, it will be necessary to educate the public about the use of PGPB in agriculture on a large scale. Much popular mythology is directed toward thinking about bacteria only as agents of disease. This misconception needs to be corrected before the public accepts the deliberate release of beneficial bacteria into the environment on a large scale. Third, while initial PGPB are likely to be nontransformed bacterial strains that have been selected for certain positive traits, it is likely in the future, as a greater understanding of the mechanisms at play in the bacterial stimulation of plant growth is gained, that scientists will genetically engineer more efficacious strains. Scientists will need to prove to both the public and to regulatory agencies worldwide that genetically engineered PGPB do not present any new hazards or risks. Fourth, scientists will need to determine whether future research should be directed toward developing PGPB that are rhizospheric or endophytic. Fifth, it will be necessary to better understand and then to optimize the relationship between PGPB and mychorrhizae [109].

Notwithstanding the above-mentioned constraints, there is every reason to believe that agricultural practice will slowly be able to shift its focus to the efficacious use of PGPB. Thus, the future of this technology looks extremely bright. 

## References

[1] Lucy M, Reed E, Glick BR (2004). Applications of free living plant growth-promoting rhizobacteria. *Antonie van Leeuwenhoek*.

[2] de Rosa CT, Johnson BL, Fay M, Hansen H, Mumtaz MM (1996). Public health implications of hazardous waste sites: findings, assessment and research. *Food and Chemical Toxicology*.

[3] Glick BR (2010). Using soil bacteria to facilitate phytoremediation. *Biotechnology Advances*.

[4] Ziegler J (1993). Health risk assessment research: the OTA report. *Environmental Health Perspectives*.

[5] Alkorta I, Garbisu C (2001). Phytoremediation of organic contaminants in soils. *Bioresource Technology*.

[6] Pilon-Smits E (2005). Phytoremediation. *Annual Review of Plant Biology*.

[7] Pilon-Smits E, Freeman JL (2006). Environmental cleanup using plants: biotechnological advances and ecological considerations. *Frontiers in Ecology and the Environment*.

[8] Salt DE, Blaylock M, Kumar NPBA (1995). Phytoremediation: a novel strategy for the removal of toxic metals from the environment using plants. *Nature Biotechnology*.

[9] Gamalero E, Glick BR, Anjum NA, Pereira ME, Ahmad I, Duarte AC, Umar S, Khan NA (2012). Plant growth-promoting bacteria and metal phytoremediation. *Phytotechnologies*.

[10] Glick BR, Stearns JC (2011). Making phytoremediation work better: maximizing a plant’s growth potential in the midst of adversity. *International Journal of Phytoremediation*.

[11] Schoenborn L, Yates PS, Grinton BE, Hugenholtz P, Janssen PH (2004). Liquid serial dilution is inferior to solid media for isolation of cultures representative of the phylum-level diversity of soil bacteria. *Applied and Environmental Microbiology*.

[12] Timmusk S, Paalme V, Pavlicek T (2011). Bacterial distribution in the rhizosphere of wild barley under contrasting microclimates. *PLoS One*.

[13] Glick BR, Patten CL, Holguin G, Penrose DM (1999). *Biochemical and Genetic Mechanisms Used by Plant Growth Promoting Bacteria*.

[14] Badri DV, Weir TL, van der Lelie D, Vivanco JM (2009). Rhizosphere chemical dialogues: plant-microbe interactions. *Current Opinion in Biotechnology*.

[15] Badri DV, Vivanco JM (2009). Regulation and function of root exudates. *Plant, Cell and Environment*.

[16] Bais HP, Weir TL, Perry LG, Gilroy S, Vivanco JM (2006). The role of root exudates in rhizosphere interactions with plants and other organisms. *Annual Review of Plant Biology*.

[17] Whipps JM, Lynch JM (1990). Carbon utilization. *The Rhizosphere,*.

[18] Lynch JM (1990). *The Rhizosphere*.

[19] Dubeikovsky AN, Mordukhova EA, Kochetkov VV, Polikarpova FY, Boronin AM (1993). Growth promotion of blackcurrant softwood cuttings by recombinant strain *Pseudomonas fluorescens* BSP53a synthesizing an increased amount of indole-3-acetic acid. *Soil Biology and Biochemistry*.

[20] Glick BR (1995). The enhancement of plant growth by free-living bacteria. *Canadian Journal of Microbiology*.

[21] Dixon ROD, Wheeler CT (1986). *Nitrogen Fixation in Plants*.

[22] Fischer HM (1994). Genetic regulation of nitrogen fixation in rhizobia. *Microbiological Reviews*.

[23] Long SR, Buikema WJ, Ausubel FM (1982). Cloning of *Rhizobium meliloti* nodulation genes by direct complementation of Nod^−^ mutants. *Nature*.

[24] Kloepper JW, Lifshitz R, Zablotowicz RM (1989). Free-living bacterial inocula for enhancing crop productivity. *Trends in Biotechnology*.

[25] Banerjee MR, Yesmin L, Vessey JK, Rai MK (2006). Plant-growth-promoting rhizobacteria as biofertilizers and biopesticides. *Handbook of Microbial Biofertilizers*.

[26] Bashan Y, Levanony H (1990). Current status of *Azospirillum* inoculation technology: *Azospirillum* as a challenge for agriculture. *Canadian Journal of Microbiology*.

[27] James EK, Olivares FL (1997). Infection and colonization of sugar cane and other graminaceous plants by endophytic diazotrophs. *Critical Reviews in Plant Sciences*.

[28] Marroquí S, Zorreguieta A, Santamaría C (2001). Enhanced symbiotic performance by *Rhizobium tropici* glycogen synthase mutants. *Journal of Bacteriology*.

[29] Ramírez M, Valderrama B, Arredondo-Peter R, Soberón M, Mora J, Hernández G (1999). *Rhizobium etli* genetically engineered for the heterologous expression of *Vitreoscilla* sp. hemoglobin: effects on free-living and symbiosis. *Molecular Plant-Microbe Interactions*.

[30] Ma W, Penrose DM, Glick BR (2002). Strategies used by rhizobia to lower plant ethylene levels and increase nodulation. *Canadian Journal of Microbiology*.

[31] Yuhashi KI, Ichikawa N, Ezura H (2000). Rhizobitoxine production by *Bradyrhizobium elkanii* enhances nodulation and competitiveness on *Macroptilium atropurpureum*. *Applied and Environmental Microbiology*.

[32] Ma W, Guinel FC, Glick BR (2003). *Rhizobium leguminosarum* biovar viciae 1-aminocyclopropane-1-carboxylate deaminase promotes nodulation of pea plants. *Applied and Environmental Microbiology*.

[33] Ma W, Charles TC, Glick BR (2004). Expression of an exogenous 1-aminocyclopropane-1-carboxylate deaminase gene in *Sinorhizobium meliloti* increases its ability to nodulate alfalfa. *Applied and Environmental Microbiology*.

[34] Duan J, Müller KM, Charles TC, Vesely S, Glick BR (2009). 1-Aminocyclopropane-1-carboxylate (ACC) deaminase genes in rhizobia from southern saskatchewan. *Microbial Ecology*.

[35] Khan MS, Zaidi A, Wani PA (2007). Role of phosphate-solubilizing microorganisms in sustainable agriculture—a review. *Agronomy for Sustainable Development*.

[36] Feng K, Lu HM, Sheng HJ, Wang XL, Mao J (2004). Effect of organic ligands on biological availability of inorganic phosphorus in soils. *Pedosphere*.

[37] Richardson AE (2001). Prospects for using soil microorganisms to improve the acquisition of phosphorus by plants. *Functional Plant Biology*.

[38] Rodríguez H, Fraga R (1999). Phosphate solubilizing bacteria and their role in plant growth promotion. *Biotechnology Advances*.

[39] Bnayahu BY, Waisel Y, Eshel A, Kafkafi U (1991). Root excretions and their environmental effects: influence on availability of phosphorus. *Plant Roots: The Hidden Half*.

[40] Rodriguez H, Gonzalez T, Goire I, Bashan Y (2004). Gluconic acid production and phosphate solubilization by the plant growth-promoting bacterium *Azospirillum* spp. *Naturwissenschaften*.

[41] Tao GC, Tian SJ, Cai MY, Xie GH (2008). Phosphate-solubilizing and -mineralizing abilities of bacteria isolated from soils1 1 project supported by the Scientific Research Foundation for the Returned Overseas Chinese Scholars, the Ministry of Education of the P.R. China. *Pedosphere*.

[42] Rojas A, Holguin G, Glick BR, Bashan Y (2001). Synergism between *Phyllobacterium* sp. (N2-fixer) and *Bacillus licheniformis* (P-solubilizer), both from a semiarid mangrove rhizosphere. *FEMS Microbiology Ecology*.

[43] Ma JF (2005). Plant root responses to three abundant soil minerals: silicon, aluminum and iron. *Critical Reviews in Plant Sciences*.

[44] Guerinot ML, Ying Y (1994). Iron: nutritious, noxious, and not readily available. *Plant Physiology*.

[45] Loper JE, Buyer JS (1991). Siderophores in microbial interactions on plant surfaces. *Molecular Plant-Microbe Interactions*.

[46] Hider RC, Kong X (2010). Chemistry and biology of siderophores. *Natural Product Reports*.

[47] Neilands JB (1981). Iron absorption and transport in microorganisms. *Annual Review of Nutrition*.

[48] Crowley DE, Reid CPP, Szaniszlo PJ (1988). Utilization of microbial siderophores in iron acquisition by oat. *Plant Physiology*.

[49] Duijff BJ, Bakker PAHM, Schippers B (1994). Ferric pseudobactin 358 as an iron source for carnation. *Journal of Plant Nutrition*.

[50] Duijff BJ, de Kogel WJ, Bakker PAHM, Schippers B (1994). Influence of pseudobactin 358 on the iron nutrition of barley. *Soil Biology and Biochemistry*.

[51] Jin CW, He YF, Tang CX, Wu P, Zheng SJ (2006). Mechanisms of microbially enhanced Fe acquisition in red clover (*Trifolium pratense* L.). *Plant, Cell and Environment*.

[52] Robin A, Vansuyt G, Hinsinger P, Meyer JM, Briat JF, Lemanceau P (2008). Iron dynamics in the rhizosphere. Consequences for plant health and nutrition. *Advances in Agronomy*.

[53] Siebner-Freibach H, Hadar Y, Chen Y (2003). Siderophores sorbed on Ca-montmorillonite as an iron source for plants. *Plant and Soil*.

[54] Walter A, Römheld V, Marschner H, Crowley DE (1994). Iron nutrition of cucumber and maize: effect of *Pseudomonas putida* YC 3 and its siderophore. *Soil Biology and Biochemistry*.

[55] Yehuda Z, Shenker M, Römheld V, Marschner H, Hadar Y, Chen Y (1996). The role of ligand exchange in the uptake of iron from microbial siderophores by gramineous plants. *Plant Physiology*.

[56] Sharma A, Johri BN, Sharma AK, Glick BR (2003). Plant growth-promoting bacterium *Pseudomonas* sp. strain GRP3 influences iron acquisition in mung bean (*Vigna radiata* L. Wilzeck). *Soil Biology and Biochemistry*.

[57] Vansuyt G, Robin A, Briat JF, Curie C, Lemanceau P (2007). Iron acquisition from Fe-pyoverdine by *Arabidopsis thaliana*. *Molecular Plant-Microbe Interactions*.

[58] Belimov AA, Hontzeas N, Safronova VI (2005). Cadmium-tolerant plant growth-promoting bacteria associated with the roots of Indian mustard (*Brassica juncea* L. Czern.). *Soil Biology and Biochemistry*.

[59] Braud A, Jézéquel K, Vieille E, Tritter A, Lebeau T (2006). Changes in extractability of Cr and Pb in a polycontaminated soil after bioaugmentation with microbial producers of biosurfactants, organic acids and siderophores. *Water, Air, and Soil Pollution*.

[60] Burd GI, Dixon DG, Glick BR (1998). A plant growth-promoting bacterium that decreases nickel toxicity in seedlings. *Applied and Environmental Microbiology*.

[61] Burd GI, Dixon DG, Glick BR (2000). Plant growth-promoting bacteria that decrease heavy metal toxicity in plants. *Canadian Journal of Microbiology*.

[62] Diels L, van der Lelie N, Bastiaens L (2002). New developments in treatment of heavy metal contaminated soils. *Reviews in Environmental Science and Biotechnology*.

[63] Robin A, Mougel C, Siblot S, Vansuyt G, Mazurier S, Lemanceau P (2006). Effect of ferritin overexpression in tobacco on the structure of bacterial and pseudomonad communities associated with the roots. *FEMS Microbiology Ecology*.

[64] Davies PJ (2004). *Plant Hormones: Biosynthesis, Signal Transduction, Action!*.

[65] Glick BR, Cheng Z, Czarny J, Duan J (2007). Promotion of plant growth by ACC deaminase-producing soil bacteria. *European Journal of Plant Pathology*.

[66] Salamone IEG, Hynes RK, Nelson LM, Siddiqui ZA (2005). Role of cytokinins in plant growth promotion by rhizosphere bacteria. *PGPR: Biocontrol and Biofertilization*.

[67] Nieto KF, Frankenberger WT (1989). Biosynthesis of cytokinins by *Azotobacter chroococcum*. *Soil Biology and Biochemistry*.

[68] Salamone IEG, Hynes RK, Nelson LM (2001). Cytokinin production by plant growth promoting rhizobacteria and selected mutants. *Canadian Journal of Microbiology*.

[69] Taller BJ, Wong TY (1989). Cytokinins in *Azotobacter vinelandii* culture medium. *Applied and Environmental Microbiology*.

[70] Tien T, Gaskin M, Hubbel D (1979). Plant growth substances produced by *Azospirillum brasilense* and their effect on the growth of pearl millet (*Pennisetum americanum* L.). *Applied and Environmental Microbiology*.

[71] Timmusk S, Nicander B, Granhall U, Tillberg E (1999). Cytokinin production by *Paenibacillus polymyxa*. *Soil Biology and Biochemistry*.

[72] Williams PM, de Mallorca MS (1982). Abscisic acid and gibberellin-like substances in roots and root nodules of *Glycine max*. *Plant and Soil*.

[73] Atzorn R, Crozier A, Wheeler CT, Sandberg G (1988). Production of gibberellins and indole-3-acetic acid by *Rhizobium phaseoli* in relation to nodulation of *Phaseolus vulgaris* roots. *Planta*.

[74] Joo GJ, Kim YM, Kim JT, Rhee IK, Kim JH, Lee IJ (2005). Gibberellins-producing rhizobacteria increase endogenous gibberellins content and promote growth of red peppers. *Journal of Microbiology*.

[75] Kang SM, Joo GJ, Hamayun M (2009). Gibberellin production and phosphate solubilization by newly isolated strain of *Acinetobacter calcoaceticus* and its effect on plant growth. *Biotechnology Letters*.

[76] Lorteau MA, Ferguson BJ, Guinel FC (2001). Effects of cytokinin on ethylene production and nodulation in pea (*Pisum sativum*) cv. Sparkle. *Physiologia Plantarum*.

[77] Yahalom E, Okon Y, Dovrat A (1990). Possible mode of action of *Azospirillum brasilense* strain Cd on the root morphology and nodule formation in burr medic (*Medicago polymorpha*). *Canadian Journal of Microbiology*.

[78] Patten CL, Glick BR (1996). Bacterial biosynthesis of indole-3-acetic acid. *Canadian Journal of Microbiology*.

[79] Spaepen S, Vanderleyden J, Remans R (2007). Indole-3-acetic acid in microbial and microorganism-plant signaling. *FEMS Microbiology Reviews*.

[80] Spaepen S, Vanderleyden J (2011). Auxin and plant-microbe interactions. *Cold Spring Harbor perspectives in biology*.

[81] Tsavkelova EA, Klimova SY, Cherdyntseva TA, Netrusov AI (2006). Microbial producers of plant growth stimulators and their practical use: a review. *Applied Biochemistry and Microbiology*.

[82] Pilet PE, Saugy M (l987). Effect on root growth of endogenous and applied IAA and ABA. *Plant Physiology*.

[83] Patten CL, Glick BR (2002). Role of *Pseudomonas putida* indoleacetic acid in development of the host plant root system. *Applied and Environmental Microbiology*.

[84] Xie H, Pasternak JJ, Glick BR (1996). Isolation and characterization of mutants of the plant growth-promoting rhizobacterium *Pseudomonas putida* GR12-2 that overproduce indoleacetic acid. *Current Microbiology*.

[85] Mayak S, Tirosh T, Glick BR (1999). Effect of wild-type and mutant plant growth-promoting rhizobacteria on the rooting of mung bean cuttings. *Journal of Plant Growth Regulation*.

[86] Jackson MB, Mattoo AK, Suttle JC (1991). Ethylene in root growth and development. *The Plant Hormone Ethylene*.

[87] Riov J, Yang SF (1989). Ethylene and auxin-ethylene interaction in adventitious root formation in mung bean (*Vigna radiata*) cuttings. *Journal of Plant Growth Regulation*.

[88] Badenoch-Jones J, Summons RE, Rolfe BG, Letham DS (1984). Phytohormones, *Rhizobium* mutants, and nodulation in legumes. IV. Auxin metabolites in pea root nodules. *Journal of Plant Growth Regulation*.

[89] Mathesius U, Schlaman HRM, Spaink HP, Sautter C, Rolfe BG, Djordjevic MA (1998). Auxin transport inhibition precedes root nodule formation in white clover roots and is regulated by flavonoids and derivatives of chitin oligosaccharides. *Plant Journal*.

[90] Fukuhara H, Minakawa Y, Akao S, Minamisawa K (1994). The involvement of indole-3-acetic acid produced by *Bradyrhizobium elkanii* in nodule formation. *Plant and Cell Physiology*.

[91] Theunis M (2005). *IAA biosynthesis in rhizobia and its potential role in symbiosis [Ph.D. thesis]*.

[92] Abeles FB, Morgan PW, Saltveit ME (1992). *Ethylene in Plant Biology*.

[93] Morgan PW, Drew MC (1997). Ethylene and plant responses to stress. *Physiologia Plantarum*.

[94] Ciardi JA, Tieman DM, Lund ST, Jones JB, Stall RE, Klee HJ (2000). Response to *Xanthomonas campestris* pv. *vesicatoria* in tomato involves regulation of ethylene receptor gene expression. *Plant Physiology*.

[95] Honma M, Shimomura T (1978). Metabolism of 1-aminocyclopropane-1-carboxylic acid. *Agricultural and Biological Chemistry*.

[96] Blaha D, Prigent-Combaret C, Mirza MS, Moënne-Loccoz Y (2006). Phylogeny of the 1-aminocyclopropane-1-carboxylic acid deaminase-encoding gene acdS in phytobeneficial and pathogenic Proteobacteria and relation with strain biogeography. *FEMS Microbiology Ecology*.

[97] Glick BR, Todorovic B, Czarny J, Cheng Z, Duan J, McConkey B (2007). Promotion of plant growth by bacterial ACC deaminase. *Critical Reviews in Plant Sciences*.

[98] Glick BR, Penrose DM, Li J (1998). A model for the lowering of plant ethylene concentrations by plant growth-promoting bacteria. *Journal of Theoretical Biology*.

[99] Penrose DM, Glick BR (2001). Levels of ACC and related compounds in exudate and extracts of canola seeds treated with ACC deaminase-containing plant growth-promoting bacteria. *Canadian Journal of Microbiology*.

[100] Contesto C, Desbrosses G, Lefoulon C (2008). Effects of rhizobacterial ACC deaminase activity on *Arabidopsis* indicate that ethylene mediates local root responses to plant growth-promoting rhizobacteria. *Plant Science*.

[101] Dey R, Pal KK, Bhatt DM, Chauhan SM (2004). Growth promotion and yield enhancement of peanut (*Arachis hypogaea* L.) by application of plant growth-promoting rhizobacteria. *Microbiological Research*.

[102] Hall JA, Peirson D, Ghosh S, Glick BR (1996). Root elongation in various agronomic crops by the plant growth promoting rhizobacterium *Pseudomonas putida* GR12-2. *Israel Journal of Plant Sciences*.

[103] Nascimento F, Brígido C, Alho L, Glick BR, Oliveira S (2012). Enhanced chickpea growth promotion ability of a mesorhizobia expressing an exogenous ACC deaminase gene. *Plant and Soil*.

[104] Naveed M, Zahir ZA, Khalid M, Asghar HN, Akhtar MJ, Arshad M (2008). Rhizobacteria containing acc-deaminase for improving growth and yield of wheat under fertilized conditions. *Pakistan Journal of Botany*.

[105] Onofre-Lemus J, Hernández-Lucas I, Girard L, Caballero-Mellado J (2009). ACC (1-aminocyclopropane-1-carboxylate) deaminase activity, a widespread trait in *Burkholderia* species, and its growth-promoting effect on tomato plants. *Applied and Environmental Microbiology*.

[106] Shah S, Li J, Moffatt BA, Glick BR (1998). Isolation and characterization of ACC deaminase genes from two different plant growth-promoting rhizobacteria. *Canadian Journal of Microbiology*.

[107] Shaharoona B, Arshad M, Zahir ZA (2006). Effect of plant growth promoting rhizobacteria containing ACC-deaminase on maize (*Zea mays* L.) growth under axenic conditions and on nodulation in mung bean (*Vigna radiata* L.). *Letters in Applied Microbiology*.

[108] Nascimento FX, Brígido C, Glick BR, Oliveira S, Alho L (2012). *Mesorhizobium ciceri* LMS-1 expressing an exogenous ACC deaminase increases its nodulation abilities and chickpea plant resistance to soil constraints. *Letters in Applied Microbiology*.

[109] Gamalero E, Berta G, Massa N, Glick BR, Lingua G (2008). Synergistic interactions between the ACC deaminase-producing bacterium *Pseudomonas putida* UW4 and the AM fungus *Gigaspora rosea* positively affect cucumber plant growth. *FEMS Microbiology Ecology*.

[110] Compant S, Duffy B, Nowak J, Clément C, Ait Barka E (2005). Use of plant growth-promoting bacteria for biocontrol of plant diseases: principles, mechanisms of action, and future prospects. *Applied and Environmental Microbiology*.

[111] Haas D, Keel C (2003). Regulation of antibiotic production in root-colonizing *Pseudomonas* spp. and relevance for biological control of plant disease. *Annual Review of Phytopathology*.

[112] Mazurier S, Corberand T, Lemanceau P, Raaijmakers JM (2009). Phenazine antibiotics produced by fluorescent pseudomonads contribute to natural soil suppressiveness to *Fusarium* wilt. *ISME Journal*.

[113] Pal KK, McSpadden Gardener B Biological control of plant pathogens. http://www.apsnet.org/edcenter/advanced/topics/Documents/PHI-BiologicalControl.pdf.

[114] Raaijmakers JM, Vlami M, de Souza JT (2002). Antibiotic production by bacterial biocontrol agents. *Antonie van Leeuwenhoek*.

[115] Whipps JM (2001). Microbial interactions and biocontrol in the rhizosphere. *Journal of Experimental Botany*.

[116] Frankowski J, Lorito M, Scala F, Schmid R, Berg G, Bahl H (2001). Purification and properties of two chitinolytic enzymes of *Serratia plymuthica* HRO-C48. *Archives of Microbiology*.

[117] Kim YC, Jung H, Kim KY, Park SK (2008). An effective biocontrol bioformulation against *Phytophthora* blight of pepper using growth mixtures of combined chitinolytic bacteria under different field conditions. *European Journal of Plant Pathology*.

[118] Ordentlich A, Elad Y, Chet I (1988). The role of chitinase of Serratia marcescens in biocontrol of Sclerotium rolfsii. *Phytopathol*.

[119] Singh PP, Shin YC, Park CS, Chung YR (1999). Biological control of *Fusarium* wilt of cucumber by chitinolytic bacteria. *Phytopathology*.

[120] Dowling DN, Sexton R, Fenton A, Nakazawa T, Furukawa K, Haas D, Silver S (1996). Iron regulation in plant-associated *Pseudomonas fluorescens* M114: implications for biological control. *Molecular Biology of Pseudomonads*.

[121] Kloepper JW, Leong J, Teintze M, Schroth MN (1980). Enhanced plant growth by siderophores produced by plant growth-promoting rhizobacteria. *Nature*.

[122] Schippers B, Bakker AW, Bakker AHM (1987). Interactions of deleterious and beneficial rhizosphere microorganisms and the effect of cropping practice. *Annual Review of Phytopathology*.

[123] O’Sullivan DJ, O’Gara F (1992). Traits of fluorescent *Pseudomonas* spp. involved in suppression of plant root pathogens. *Microbiological Reviews*.

[124] Bar-Ness E, Chen Y, Hadar Y, Marschner H, Römheld V, Chen Y, Hadar Y (1991). Siderophores of *Pseudomonas putida* as an iron source for dicot and monocot plants. *Iron Nutrition and Interactions in Plants,*.

[125] Wang Y, Brown HN, Crowley DE, Szaniszlo PJ (1993). Evidence for direct utilization of a siderophore, ferrioxamine B, in axenically grown cucumber. *Plant, Cell & Environment*.

[126] Buysens S, Heungens K, Poppe J, Höfte M (1996). Involvement of pyochelin and pyoverdin in suppression of *Pythium*-induced damping-off of tomato by *Pseudomonas aeruginosa* 7NSK2. *Applied and Environmental Microbiology*.

[127] Elsherif M, Grossmann F (1994). Comparative investigations on the antagonistic activity of fluorescent pseudomonads against *Gaeumannomyces graminis* var. tritici *in vitro* and *in vivo*. *Microbiological Research*.

[128] Martinetti G, Loper JE (1992). Mutational analysis of genes determining antagonism of *Alcaligenes* sp. strain MFA1 against the phytopathogenic fungus *Fusarium oxysporum*. *Canadian Journal of Microbiology*.

[129] Vandenbergh PA, Gonzalez CF Method for protecting the growth of plants employing mutant siderophore producing strains of *Pseudomonas putida*.

[130] Innerebner G, Knief C, Vorholt JA (2011). Protection of *Arabidopsis thaliana* against leaf-pathogenic *Pseudomonas syringae* by *Sphingomonas* strains in a controlled model system. *Applied and Environmental Microbiology*.

[131] Glick BR, Bashan Y (1997). Genetic manipulation of plant growth-promoting bacteria to enhance biocontrol of phytopathogens. *Biotechnology Advances*.

[132] Hao Y, Charles TC, Glick BR (2007). ACC deaminase from plant growth-promoting bacteria affects crown gall development. *Canadian Journal of Microbiology*.

[133] Hao Y, Charles TC, Glick BR (2011). An ACC deaminase containing *A. tumefaciens* strain D3 shows biocontrol activity to crown gall disease. *Canadian Journal of Microbiology*.

[134] Husen E, Wahyudi AT, Suwanto A, Giyanto (2011). Growth enhancement and disease reduction of soybean by 1-aminocyclopropane-1-carboxylate deaminase-producing Pseudomonas. *American Journal of Applied Sciences*.

[135] Toklikishvili N, Dandurishvili N, Tediashvili M (2010). Inhibitory effect of ACC deaminase-producing bacteria on crown gall formation in tomato plants infected by *Agrobacterium tumefaciens* or *A. vitis*. *Plant Pathology*.

[136] Wang C, Knill E, Glick BR, Défago G (2000). Effect of transferring 1-aminocyclopropane-1-carboxylic acid (ACC) deaminase genes into *Pseudomonas fluorescens* strain CHA0 and its *gacA* derivative CHA96 on their growth-promoting and disease-suppressive capacities. *Canadian Journal of Microbiology*.

[137] Lund ST, Stall RE, Klee HJ (1998). Ethylene regulates the susceptible response to pathogen infection in tomato. *Plant Cell*.

[138] Robison MM, Shah S, Tamot B, Pauls KP, Moffatt BA, Glick BR (2001). Reduced symptoms of Verticillium wilt in transgenic tomato expressing a bacterial ACC deaminase. *Molecular Plant Pathology*.

[139] Pieterse CMJ, Leon-Reyes A, van der Ent S, van Wees SCM (2009). Networking by small-molecule hormones in plant immunity. *Nature Chemical Biology*.

[140] Verhagen BWM, Glazebrook J, Zhu T, Chang HS, van Loon LC, Pieterse CMJ (2004). The transcriptome of rhizobacteria-induced systemic resistance in *Arabidopsis*. *Molecular Plant-Microbe Interactions*.

[141] Bakker PAHM, Pieterse CMJ, van Loon LC (2007). Induced systemic resistance by fluorescent *Pseudomonas* spp. *Phytopathology*.

[142] Glick BR (2004). Bacterial ACC deaminase and the alleviation of plant stress. *Advances in Applied Microbiology*.

[143] Glick BR, Liu C, Ghosh S, Dumbroff EB (1997). Early development of canola seedlings in the presence of the plant growth-promoting rhizobacterium *Pseudomonas putida* GR12-2. *Soil Biology and Biochemistry*.

[144] Grichko VP, Glick BR (2001). Amelioration of flooding stress by ACC deaminase-containing plant growth-promoting bacteria. *Plant Physiology and Biochemistry*.

[145] Mayak S, Tirosh T, Glick BR (2004). Plant growth-promoting bacteria that confer resistance to water stress in tomatoes and peppers. *Plant Science*.

[146] Zahir ZA, Munir A, Asghar HN, Shaharoona B, Arshad M (2008). Effectiveness of rhizobacteria containing ACC deaminase for growth promotion of peas (*Pisum sativum*) under drought conditions. *Journal of Microbiology and Biotechnology*.

[147] Cheng Z, Wei YYC, Sung WWL, Glick BR, McConkey BJ (2009). Proteomic analysis of the response of the plant growth-promoting bacterium *Pseudomonas putida* UW4 to nickel stress. *Proteome Science*.

[148] Farwell AJ, Vesely S, Nero V (2006). The use of transgenic canola (*Brassica napus*) and plant growth-promoting bacteria to enhance plant biomass at a nickel-contaminated field site. *Plant and Soil*.

[149] Nie L, Shah S, Rashid A, Burd GI, Dixon DG, Glick BR (2002). Phytoremediation of arsenate contaminated soil by transgenic canola and the plant growth-promoting bacterium *Enterobacter cloacae* CAL2. *Plant Physiology and Biochemistry*.

[150] Reed MLE, Glick BR (2005). Growth of canola (*Brassica napus*) in the presence of plant growth-promoting bacteria and either copper or polycyclic aromatic hydrocarbons. *Canadian Journal of Microbiology*.

[151] Reed MLE, Warner BG, Glick BR (2005). Plant growth-promoting bacteria facilitate the growth of the common reed *Phragmites australis* in the presence of copper or polycyclic aromatic hydrocarbons. *Current Microbiology*.

[152] Safronova VI, Stepanok VV, Engqvist GL, Alekseyev YV, Belimov AA (2006). Root-associated bacteria containing 1-aminocyclopropane-1-carboxylate deaminase improve growth and nutrient uptake by pea genotypes cultivated in cadmium supplemented soil. *Biology and Fertility of Soils*.

[153] Li J, Sun J, Yang Y, Guo S, Glick BR (2012). Identification of hypoxic-responsive proteins in cucumber using a proteomic approach. *Plant Physiology and Biochemistry*.

[154] Ahmad M, Zahir ZA, Asghar HN, Asghar M (2011). Inducing salt tolerance in mung bean through coinoculation with rhizobia and plant-growthpromoting rhizobacteria containing 1-aminocyclopropane-1-carboxylate deaminase. *Canadian Journal of Microbiology*.

[155] Cheng Z, Park E, Glick BR (2007). 1-Aminocyclopropane-1-carboxylate deaminase from *Pseudomonas putida* UW4 facilitates the growth of canola in the presence of salt. *Canadian Journal of Microbiology*.

[156] Cheng Z, Woody OZ, McConkey BJ, Glick BR (2012). Combined effects of the plant growth-promoting bacterium *Pseudomonas putida* UW4 and salinity stress on the *Brassica napus* proteome. *Applied Soil Ecology*.

[157] Gamalero E, Berta G, Massa N, Glick BR, Lingua G (2010). Interactions between *Pseudomonas putida* UW4 and *Gigaspora rosea* BEG9 and their consequences for the growth of cucumber under salt-stress conditions. *Journal of Applied Microbiology*.

[158] Jalili F, Khavazi K, Pazira E (2009). Isolation and characterization of ACC deaminase-producing fluorescent pseudomonads, to alleviate salinity stress on canola (*Brassica napus* L.) growth. *Journal of Plant Physiology*.

[159] Mayak S, Tirosh T, Glick BR (2004). Plant growth-promoting bacteria confer resistance in tomato plants to salt stress. *Plant Physiology and Biochemistry*.

[160] Nadeem SM, Zahir ZA, Naveed M, Arshad M (2007). Preliminary investigations on inducing salt tolerance in maize through inoculation with rhizobacteria containing ACC deaminase activity. *Canadian Journal of Microbiology*.

[161] Sadrnia M, Maksimava N, Khromsova E, Stanislavich S, Owilia P, Arjomandzadegan M (2011). Study the effect of bacterial 1-aminocyclopropane-1-carboxylatedeaminase (ACC deaminase) on resistance to salt stress in tomato plants. *Anals of University of Oradea*.

[162] Saravanakumar D, Samiyappan R (2007). ACC deaminase from *Pseudomonas fluorescens* mediated saline resistance in groundnut (*Arachis hypogea*) plants. *Journal of Applied Microbiology*.

[163] Siddikee MA, Chauhan PS, Anandham R, Han GH, Sa T (2010). Isolation, characterization, and use for plant growth promotion under salt stress, of ACC deaminase-producing halotolerant bacteria derived from coastal soil. *Journal of Microbiology and Biotechnology*.

[164] Siddikee MA, Glick BR, Chauhan PS, Yim WJ, Sa T (2011). Enhancement of growth and salt tolerance of red pepper seedlings (*Capsicum annuum* L.) by regulating stress ethylene synthesis with halotolerant bacteria containing 1-aminocyclopropane-1-carboxylic acid deaminase activity. *Plant Physiology and Biochemistry*.

[165] Yue HT, Mo WP, Li C, Zheng YY, Li H (2007). The salt stress relief and growth promotion effect of Rs-5 on cotton. *Plant and Soil*.

[166] Gurska J, Wang W, Gerhardt KE (2009). Three year field test of a plant growth promoting rhizobacteria enhanced phytoremediation system at a land farm for treatment of hydrocarbon waste. *Environmental Science and Technology*.

[167] Huang XD, El-Alawi Y, Penrose DM, Glick BR, Greenberg BM (2004). A multi-process phytoremediation system for removal of polycyclic aromatic hydrocarbons from contaminated soils. *Environmental Pollution*.

[168] Huang XD, El-Alawi Y, Gurska J, Glick BR, Greenberg BM (2005). A multi-process phytoremediation system for decontamination of persistent total petroleum hydrocarbons (TPHs) from soils. *Microchemical Journal*.

[169] Bianco C, Defez R (2009). *Medicago truncatula* improves salt tolerance when nodulated by an indole-3-acetic acid-overproducing *Sinorhizobium meliloti*strain. *Journal of Experimental Botany*.

[170] Bianco C, Defez R (2010). Improvement of phosphate solubilization and *Medicago* plant yield by an indole-3-acetic acid-overproducing strain of *Sinorhizobium meliloti*. *Applied and Environmental Microbiology*.

[171] de-Bashan LE, Hernandez JP, Nelson KN, Bashan Y, Maier RM (2010). Growth of quailbush in acidic, metalliferous desert mine tailings: effect of *Azospirillum brasilense* Sp6 on biomass production and Rhizosphere community structure. *Microbial Ecology*.

[172] Egamberdieva D (2009). Alleviation of salt stress by plant growth regulators and IAA producing bacteria in wheat. *Acta Physiologiae Plantarum*.

[173] Rajkumar M, Nagendran R, Lee KJ, Lee WH, Kim SZ (2006). Influence of plant growth promoting bacteria and Cr^6+^ on the growth of Indian mustard. *Chemosphere*.

[174] Sheng XF, Xia JJ (2006). Improvement of rape (*Brassica napus*) plant growth and cadmium uptake by cadmium-resistant bacteria. *Chemosphere*.

[175] Wani PA, Khan MS, Zaidi A (2007). Effect of metal tolerant plant growth promoting *Bradyrhizobium* sp. (vigna) on growth, symbiosis, seed yield and metal uptake by greengram plants. *Chemosphere*.

[176] Wani PA, Khan MS, Zaidi A (2008). Effect of metal-tolerant plant growth-promoting *Rhizobium* on the performance of pea grown in metal-amended soil. *Archives of Environmental Contamination and Toxicology*.

[177] Egamberdieva D, Kucharova Z (2009). Selection for root colonising bacteria stimulating wheat growth in saline soils. *Biology and Fertility of Soils*.

[178] Gamalero E, Glick BR, Golubev IA (2010). Bacterial ACC deaminase and IAA: interactions and consequences for plant growth in polluted environments. *Handbook of Phytoremediation*.

[179] Sgroy V, Cassán F, Masciarelli O, Del Papa MF, Lagares A, Luna V (2009). Isolation and characterization of endophytic plant growth-promoting (PGPB) or stress homeostasis-regulating (PSHB) bacteria associated to the halophyte Prosopis strombulifera. *Applied Microbiology and Biotechnology*.

[180] Czarny JC, Shah S, Glick BR, Ramina A, Chang C, Giovannoni J, Klee H, Perata P, Woltering E (2007). Response of canola plants at the transcriptional level to expression of a bacterial ACC deaminase in the roots. *Advances in Plant Ethylene Research*.

[181] Dharmasiri N, Estelle M (2004). Auxin signaling and regulated protein degradation. *Trends in Plant Science*.

[182] Stearns JC, Woody OZ, McConkey BJ, Glick BR (2012). Effects of bacterial ACC deaminase on *Brassica napus* gene expression measured with an *Arabidopsis thaliana* microarray. *Molecular Plant-Microbe Interactions*.

[183] Rivero RM, Kojima M, Gepstein A (2007). Delayed leaf senescence induces extreme drought tolerance in a flowering plant. *Proceedings of the National Academy of Sciences of the United States of America*.

[184] Rodríguez-Salazar J, Suárez R, Caballero-Mellado J, Iturriaga G (2009). Trehalose accumulation in *Azospirillum brasilense* improves drought tolerance and biomass in maize plants. *FEMS Microbiology Letters*.

[185] Suárez R, Wong A, Ramírez M (2008). Improvement of drought tolerance and grain yield in common bean by overexpressing trehalose-6-phosphate synthase in rhizobia. *Molecular Plant-Microbe Interactions*.

[186] Glick BR, Skof YC (1986). Environmental implications of recombinant DNA technology. *Biotechnology Advances*.

[187] Duman JG, Olsen TM (1993). Thermal hysteresis protein activity in bacteria, fungi, and phylogenetically diverse plants. *Cryobiology*.

[188] Sun X, Griffith M, Pasternak JJ, Glick BR (1995). Low temperature growth, freezing survival, and production of antifreeze protein by the plant growth promoting rhizobacterium *Pseudomonas putida* GR12-2. *Canadian Journal of Microbiology*.

[189] Xu H, Griffith M, Patten CL, Glick BR (1998). Isolation and characterization of an antifreeze protein with ice nucleation activity from the plant growth promoting rhizobacterium *Pseudomonas putida* GR12-2. *Canadian Journal of Microbiology*.

[190] Garnham CP, Gilbert JA, Hartman CP, Campbell RL, Laybourn-Parry J, Davies PL (2008). A Ca^2+^-dependent bacterial antifreeze protein domain has a novel *β*-helical ice-binding fold. *Biochemical Journal*.

[191] Gilbert JA, Davies PL, Laybourn-Parry J (2005). A hyperactive, Ca^2+^-dependent antifreeze protein in an Antarctic bacterium. *FEMS Microbiology Letters*.

[192] Gilbert JA, Hill PJ, Dodd CER, Laybourn-Parry J (2004). Demonstration of antifreeze protein activity in Antarctic lake bacteria. *Microbiology*.

[193] Kawahara H, Iwanaka Y, Higa S (2007). A novel, intracellular antifreeze protein in an antarctic bacterium, *Flavobacterium xanthum*. *Cryo-Letters*.

[194] Muryoi N, Sato M, Kaneko S (2004). Cloning and expression of *afpA*, a gene encoding an antifreeze protein from the arctic plant growth-promoting rhizobacterium *Pseudomonas putida* GR12-2. *Journal of Bacteriology*.

[195] Raymond JA, Fritsen C, Shen K (2007). An ice-binding protein from an Antarctic sea ice bacterium. *FEMS Microbiology Ecology*.

